# Of Mice and Monkeys: Neuroprotective Efficacy of the p38 Inhibitor BIRB 796 Depends on Model Duration in Experimental Glaucoma

**DOI:** 10.1038/s41598-020-65374-6

**Published:** 2020-05-22

**Authors:** Wendi S. Lambert, Silvia Pasini, John W. Collyer, Cathryn R. Formichella, Purnima Ghose, Brian J. Carlson, David J. Calkins

**Affiliations:** The Vanderbilt Eye Institute, Vanderbilt University Medical Center, Nashville, TN 37232-2337 USA

**Keywords:** Neurodegenerative diseases, Retina, Optic nerve diseases

## Abstract

Glaucoma is a group of optic neuropathies associated with aging and sensitivity to intraocular pressure (IOP). Early progression involves retinal ganglion cell (RGC) axon dysfunction that precedes frank degeneration. Previously we demonstrated that p38 MAPK inhibition abates axonal dysfunction and slows degeneration in the inducible microbead occlusion model of glaucoma in rat. Here, we assessed the neuroprotective effect of topical eye delivery of the p38 MAPK inhibitor BIRB 796 in three models of glaucoma (microbead occlusion in rat and squirrel monkey and the genetic DBA/2 J mouse model) with distinct durations of IOP elevation. While BIRB 796 did not influence IOP, treatment over four weeks in rats prevented degradation of anterograde axonal transport to the superior colliculus and degeneration in the optic nerve. Treatment over months in the chronic DBA/2 J model and in the squirrel monkey model reduced expression and activation of p38 downstream targets in the retina and brain but did not rescue RGC axon transport or degeneration, suggesting the efficacy of BIRB 796 in preventing associated degeneration of the RGC projection depends on the duration of the experimental model. These results emphasize the importance of evaluating potential therapeutic compounds for neuroprotection in multiple models using elongated treatment paradigms for an accurate assessment of efficacy.

## Introduction

Glaucoma is the primary source of permanent sightlessness around the world^1^, and is second only to cataract in producing vision loss. Estimates suggest that by 2020 over 75 million individuals will have glaucoma and that more than 10 million of those will already suffer from irreversible blindness^1–3^. In glaucoma, stress associated with sensitivity to intraocular pressure (IOP) is transmitted at the optic nerve head, selectively targeting retinal ganglion cells (RGCs) and their axons^4,5^. While age is a key risk factor, IOP is the only modifiable risk factor and the singular focus for clinical intervention^6^. The use of topical IOP-lowering drugs is the first line treatment for glaucoma^7^. Glaucoma progression can be slowed by reducing IOP, however RGC degeneration (and vision loss) continues for many. For nearly half the patients with glaucoma taking medications to reduce IOP, the disease will continue to worsen^6,8,9^. Neurodegeneration in glaucoma is similar to other age-related neurodegenerative disorders like amyotrophic lateral sclerosis, Parkinson’s disease, Alzheimer’s disease, and Huntington’s disease in that axonal function is compromised prior to death of neurons^10–16^. Deterioration of anterograde transport from RGCs to central targets in the brain arises early in animal models of glaucoma; degradation is followed by somatic drop-out in the retina subsequent to axonal loss in the optic nerve^17–19^. Experimental interventions that preserve axonal transport can suspend or stop subsequent degeneration of axons and cell bodies^20–22^. Given this progression, it is appropriate that the identification of neuroprotective therapies that could abate disease progression by preserving RGC structure and function independently of IOP remains a high priority^23^.

In a previous study we showed that inhibition of p38 mitogen-activated protein kinase (MAPK) with Ro3206145 protected against RGC axonal transport deficits and axon loss in rats with induced elevations in IOP via microbead occlusion of the anterior chamber^24^. The p38 MAPKs are a family of serine/threonine protein kinases that become activated in response to physical stress or injury^25–27^. Of the four p38 MAPKs identified, p38α and p38β are ubiquitously expressed, while the expression of p38γ and p38δ is tissue-specific^25^. Once activated, p38 MAPK can activate by phosphorylation downstream targets to induce changes in gene expression via transcription factor activation, inflammatory cytokine production, apoptosis, cell survival, growth and/or differentiation pathways, tissue remodeling and maintenance of the cytoskeleton^25–27^. Activation of the p38 MAPK pathway has been implicated in rheumatoid arthritis, inflammatory bowel disease, cardiovascular disease, diabetes, osteoporosis, Alzheimer’s disease, Parkinson’s disease, and glaucoma^28–34^. Due to its role in inflammation, many p38 inhibitors have been developed, and they fall into two groups based on mechanism of action: orthosteric or ATP-competitive (e.g., SB-203580, Ro3206145) and allosteric or non-competitive (e.g., BIRB 796)^35^. BIRB 796 (doramapimod) binds to p38 MAPK and causes a conformational change so that ATP cannot bind^27,35,36^. It has a dissociation rate much slower than that of other p38 inhibitors, and shows a high selectivity to and a picomolar affinity for p38, suggesting BIRB 796 would be a long-lasting drug with fewer off-target effects^27,35,36^.

Here we assessed the neuroprotective effects of BIRB 796 in three species using two models of glaucoma, the DBA/2 J mouse—an inbred strain tending towards IOP elevation with age – and the inducible microbead occlusion model in rat and squirrel monkey (SM) eyes^37–39^. The use of different species and approaches to elevate IOP in this study was deliberate, as promising neuroprotective drugs should be tested in multiple animal models to “increase the likelihood of translation to large human clinical trials”^16,40^. In this study, microbead injection into the anterior chamber of rats elevated IOP for four weeks and reduced RGC axonal transport of cholera toxin subunit B from retina to the superior colliculus in vehicle-treated rats; BIRB 796 treatment prevented this reduction and protected RGC axons from degeneration in the optic nerve. In DBA/2 J mice, IOP increased over a 24-week treatment period causing a decrease in axonal transport to the SC in vehicle-treated eyes. Similarly, microbead-induced IOP elevations in squirrel monkey eyes for 29 weeks reduced anterograde transport to the lateral geniculate nucleus (LGN). Treatment with BIRB 796 did not prevent transport degradation, nor did it protect RGC axons in the optic nerve in either model. However, treatment did reduce expression of p38 downstream targets that increased with elevated IOP, suggesting BIRB 796 did reach the retinal projection in DBA/2 J mice and SMs.

## Results

**Figure 1:**
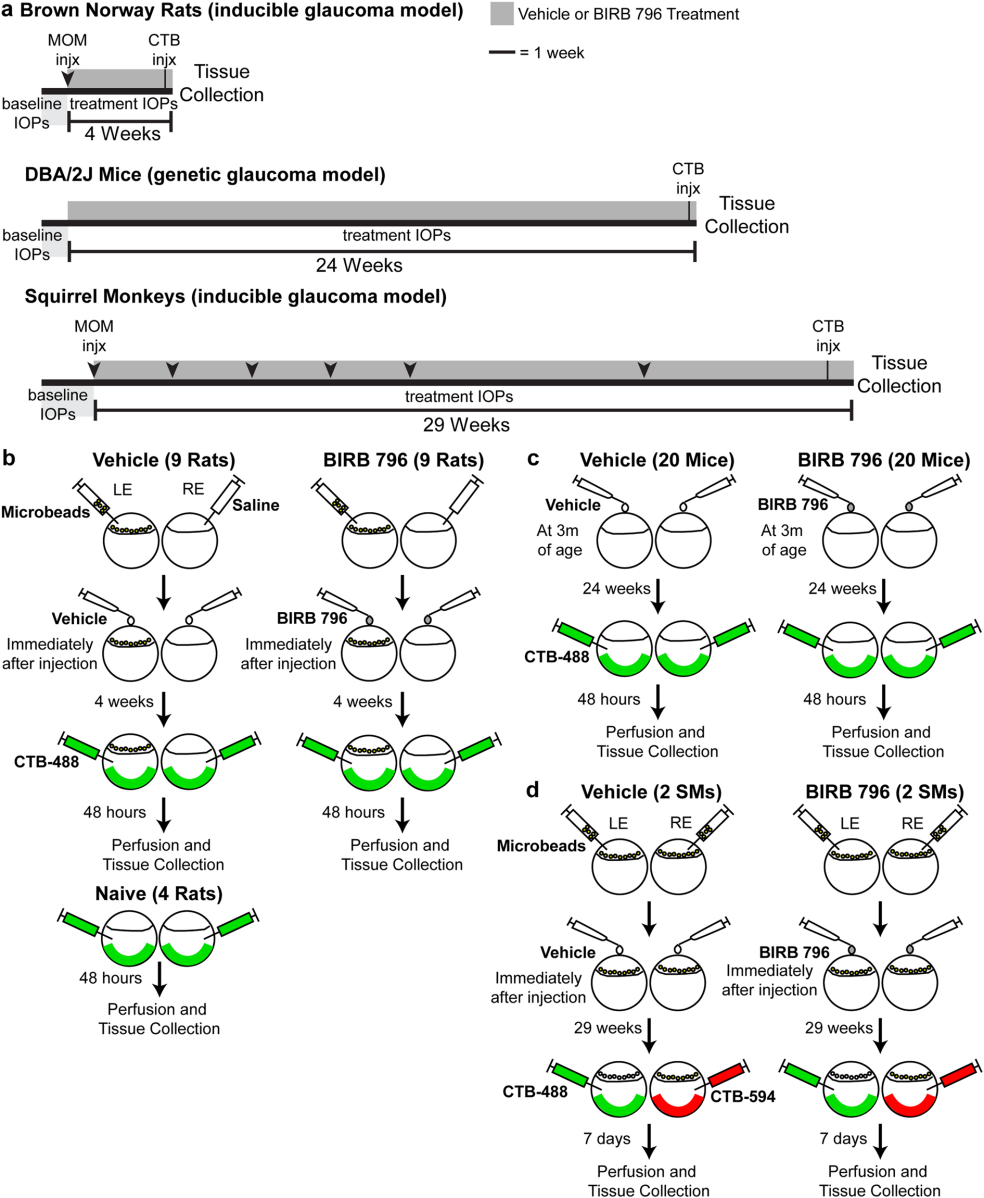
Experimental timeline and schematic. (**a**) Experimental timelines for rat, mouse and squirrel monkey cohorts. Microbead injections (MOM injx, arrowheads), intravitreal injection of cholera toxin subunit B (CTB injx, perpendicular lines), baseline intraocular pressure measurements (IOPs, light gray bars) and treatment intraocular pressure measurements (IOPs, dark gray bars) were performed according to their position on the timelines. Treatment with vehicle or BIRB 796 is indicated by the bracketed line (|—|) and length of treatment (4 weeks, 24 weeks or 29 weeks). Scale: line indicates 1 week. (**b**) Experimental schematic for rat vehicle, BIRB-796, and naïve experimental groups showing which eyes received microbead or saline injection, which eyes received vehicle or BIRB 796 treatment, and which eyes received CTB injection. (**c**) Experimental schematic for DBA/2 J mouse vehicle and BIRB-796 experimental groups indicating eyes that received vehicle or BIRB 796 treatment, and eyes received CTB injection. (**d**) Experimental schematic for squirrel monkey (SM) vehicle and BIRB-796 experimental groups showing which eyes received microbead injection, which eyes received vehicle or BIRB 796 treatment, and which eyes received CTB injection.

To examine the neuroprotective effects of BIRB 796 in glaucoma, we chose three different species (mouse, rat and non-human primate) and two different methods to elevate IOP (microbead occlusion and genetic mutation). As seen in the experimental timelines for the three models we used (Fig. 1a), the inducible microbead occlusion model in rats was of short duration (4 weeks) compared to the genetic DBA/2 J model (24 weeks) and the inducible microbead occlusion model in squirrel monkeys (29 weeks). To move BIRB 796 forward as a neuroprotective treatment for a chronic disease like glaucoma, we wanted to show efficacy over longer treatment periods and in different animal models^16,40^. Figure 1b–d illustrates the experimental methodology of microbead injections, BIRB 796 treatment, and anterograde transport tracing for each model. One rat in the vehicle group and one mouse in the vehicle group expired prior to completion of the study. No other animals, regardless of treatment, showed signs of impairment or distress (itching or rubbing of the eye, eye closure, excessive grooming of the face, Anoxeria, decreased responsiveness, labored breathing, and/or sudden lethargy) during the study.

### BIRB 796 protects anterograde transport following microbead injection in rats

**Figure 2:**
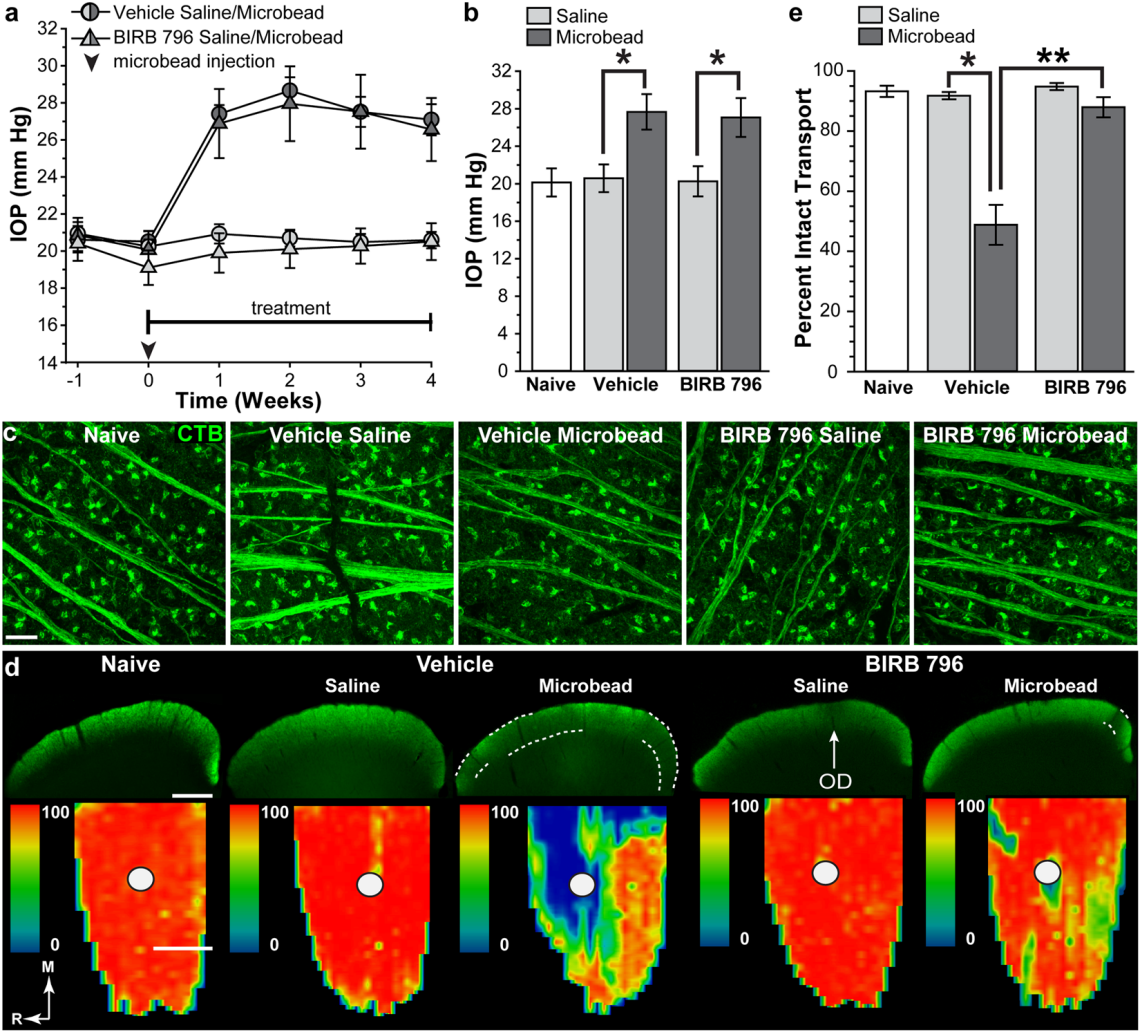
BIRB 796 protects anterograde transport in rats following IOP elevation. (**a**) Mean intraocular pressure (IOP) in rats following a single unilateral injection of microbeads into the anterior chamber (dark gray symbols). The fellow eye was injected with an equivalent volume of saline (light gray symbols). Arrowhead indicates microbead injection. (**b**) Bar graph showing mean IOP in naïve, vehicle-saline, vehicle-microbead, BIRB 796-saline and BIRB 796-microbead rat eyes. *p ≤ 0.01. (**c**) Confocal images of whole-mounted retinas showing RGC uptake and transport of CTB (green) in naïve, vehicle- and BIRB 796-treated rats. Scale: 30 μm. (**d**) Coronal sections (top row) through the superior colliculus (SC) following intravitreal injection of CTB (green) into naïve, vehicle-saline, vehicle-microbead, BIRB 796-saline and BIRB 796-microbead rat eyes. Transport deficits are indicated by dotted lines. OD: optic disc representation (arrows). Retinotopic maps (bottom row) reconstructed from serial sections of SC with optic disc indicated (circles). Density of the transported CTB signal ranges from 0% (blue) to 50% (green) to 100% (red). Medial (M) and rostral (R) orientations are indicated. Scale bars: 500 μm. (**e**) Bar graph showing mean level of intact transport to the SC from naïve, vehicle-saline, vehicle-microbead, BIRB 796-saline and BIRB 796-microbead rat eyes. *p = 0.00002; **p = 0.00007. Data expressed as mean ± SEM. Statistical comparisons made using one-way ANOVA and two-sided t-tests. n = 4 rats for naïve, 8 for vehicle, 9 for BIRB 796 groups (**a**,**b**); n = 8 SCs for naïve, vehicle-saline and vehicle-microbead and 9 SCs for BIRB 796-saline and BIRB 796-microbead groups (**d**,**e**).

Baseline IOP measurements in rats ranged from 20.1 ± 0.9 to 20.6 ± 0.5 mm Hg and were similar for vehicle and BIRB 796 groups (p = 0.7). Saline injection into the anterior chamber had no effect on IOP in vehicle-saline or BIRB 796-saline eyes, while microbead injection elevated IOP in all vehicle-microbead and BIRB 796-microbead eyes for the full 4-week treatment period (Fig. 2a). Microbead injection resulted in an elevation of 34.4% in vehicle-microbead eyes compared to vehicle-saline eyes (27.7 ± 1.9 vs. 20.6 ± 1.5 mm Hg; p = 0.005, Fig. 2b) and a 36.3% increase in BIRB 796-microbead eyes compared to BIRB 796-saline eyes (27.2 ± 2.3 vs. 19.9 ± 2.0 mm Hg; p = 0.01). Mean IOP of vehicle-saline eyes and BIRB 796-saline eyes was similar to each other and to naïve rats (20.1 ± 1.5 mm Hg; p = 0.9, Fig. 1b); IOP in BIRB 796-microbead eyes was similar to vehicle-microbead eye IOP (p = 0.8).

We evaluated transport of cholera toxin subunit B (CTB) from the retina to the superior colliculus (SC), the primary central projection for RGCs in rodents, in rats that received vehicle or BIRB 796 as anterograde axonal transport is compromised early in models of glaucoma^4,41–44^. We verified RGC uptake and initial transport of fluorescently labeled CTB in RGC axons from naïve, vehicle-saline, vehicle-microbead, BIRB 796-saline and BIRB 796-microbead retinas (Fig. 2c) and then quantified the degree of intact SC transport in retinotopic maps (Fig. 2d). Transport from vehicle-saline eyes to the SC was similar to that of BIRB 796-saline eyes and to naïve rat eyes, showing nearly complete transport across the retinotopic map (red colors).

Elevated IOP following microbead injection resulted in transport deficits in the SC of vehicle-microbead rats indicated in SC sections and by lack of representation in the retinotopic map (Fig. 2d). We observed fewer and smaller transport deficits in SCs from BIRB 796-microbead rats. When quantified (Fig. 2e), anterograde transport to the SC from BIRB 796-saline eyes (94.8 ± 3.4% intact transport) was similar to naïve eyes (93.2 ± 1.9% intact transport) and vehicle-saline eyes (91.8 ± 1.2%; p = 0.3). Transport from vehicle-microbead eyes was reduced to 48.8 ± 6.7% compared to vehicle-saline eyes (p < 0.001); BIRB 796 treatment attenuated this reduction, resulting in 87.9 ± 3.4% intact transport from BIRB 796-microbead eyes compared to BIRB 796-saline (p = 0.7). Compared to vehicle-microbead, BIRB 796 protected transport from BIRB 796-microbead eyes by 80% (p < 0.001).

### BIRB 796 protects ganglion cell axons following microbead injection in rats

**Figure 3:**
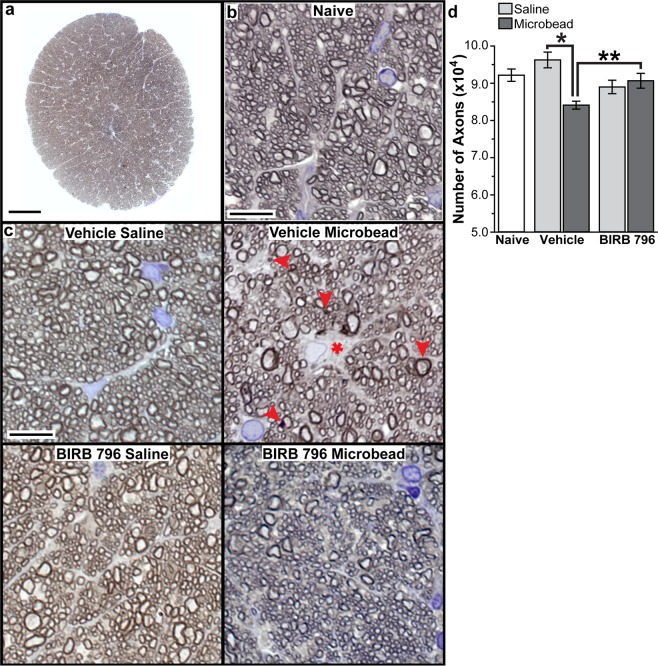
BIRB 796 protects RGC axons in rat optic nerve following IOP elevation. (**a**) Representative image of rat optic nerve montage used to count RGC axons using AxonJ^46^. Scale: 100 μm. (**b**) Representative image from a naïve rat optic nerve montage. Scale: 20 μm. (**c**) Representative images from vehicle-saline, vehicle-microbead, BIRB 796-saline and BIRB 796-microbead rat optic nerve montages. Degenerating axon profiles (arrowheads) and glial hypertrophy (asterisk) are indicated. Scale: 20 μm. (**d**) Bar graph showing mean RGC axon number from naïve, vehicle-saline, vehicle-microbead, BIRB 796-saline and BIRB 796-microbead rat optic nerve. *p = 0.0002; **p = 0.01. Data expressed as mean ± SEM. Statistical comparisons made using one-way ANOVA and two-sided t-tests. n = 8 optic nerve montages for naïve, vehicle-saline and vehicle-microbead and 9 optic nerve montages for BIRB 796-saline and BIRB 796-microbead groups.

We counted RGC axons in naïve, vehicle-saline, vehicle-microbead, BIRB 796-saline and BIRB 796-microbead optic nerves as axon degeneration in the optic nerve follows anterograde transport dysfunction to RGC targets such as the SC and LGN in models of glaucoma^4^. Figure 3a shows a representative rat optic nerve montage used to count RGC axons within the entire montage using the AxonJ macro in Fiji^45,46^. Representative images from naïve (Fig. 3b), vehicle-saline and BIRB 796-saline optic nerve montages (Fig. 3c) all appeared similar, with distinct axon profiles tightly packed into axon bundles. Vehicle-microbead optic nerves showed degenerating axon profiles and glial hypertrophy (Fig. 3c). Axons from BIRB 796-microbead optic nerves looked similar to naïve, vehicle-saline or BIRB 796-saline nerves, with very few degenerating profiles. Axon number ranged from 85,141 to 97,319 axons in naïve nerves, 87,599 to 105,553 axons in vehicle-saline nerves, and from 81,794 to 100,501 axons in BIRB 796-saline nerves. These counts are similar to previously published axons counts for rats (74,212 to 98,272 axons)^47^. Mean axon number (Fig. 3d) in vehicle-saline nerves (96,257 ± 2,113 axons) and BIRB 796-saline nerves (90,668 ± 1,996 axons) was similar to counts from naïve nerves (92,169 ± 1,658 axons; p = 0.2). Elevated IOP reduced RGC axons by 13% in vehicle-microbead nerves to an average of 84,115 ± 1,062 axons (range of 80,568 and 89,808 axons) compared to vehicle-saline nerves (p = 0.0002, Fig. 3d). In contrast, mean axon number in BIRB 796-microbead nerves (90,668 ± 1,996 axons, range of 86,223 to 103,931 axons) was similar to BIRB 796-saline nerves (p = 0.5, Fig. 3d). Compared to vehicle-microbead, BIRB 796 protected RGC axons in BIRB 796-microbead optic nerves by 8% (p = 0.01).

### BIRB 796 did not protect anterograde transport in DBA/2 J mice

**Figure 4:**
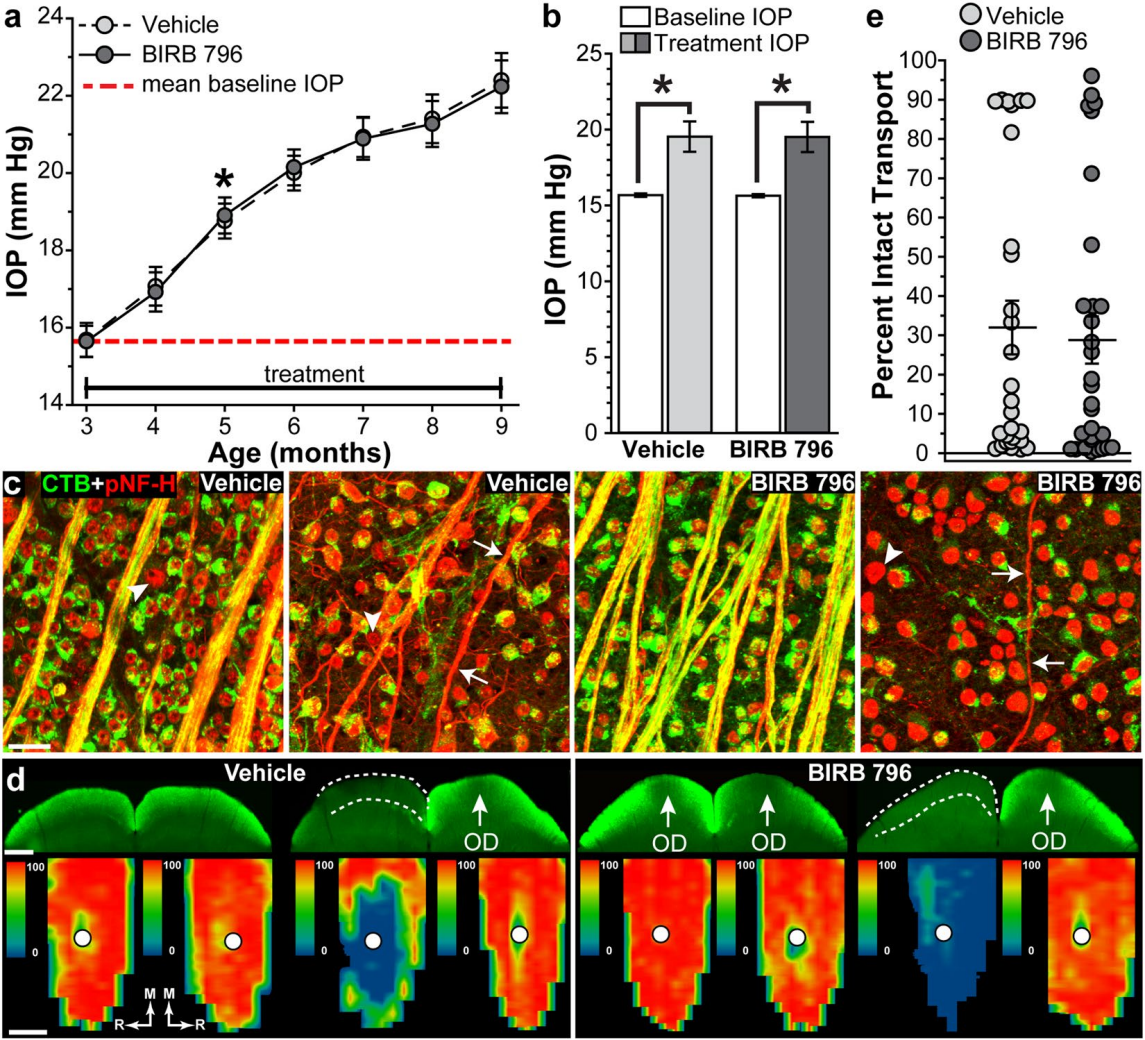
BIRB 796 and anterograde transport in DBA/2 J mice. (**a**) Mean intraocular pressure (IOP) in vehicle- and BIRB 796-treated DBA/2 J mice. Red dashed line indicates mean baseline IOP from all mice. *p ≤ 0.00002 versus baseline IOP. (**b**) Bar graph showing mean baseline IOP and treatment IOP for vehicle- and BIRB 796-treated DBA/2 J mice. *p ≤ 0.0005. (**c**) Confocal images of whole-mounted retinas showing RGC uptake and transport of CTB (green) in vehicle- and BIRB 796-treated DBA/2 J mice. Phosphorylated neurofilament-heavy (pNF-H; red) expression in RGC somas and axons is also shown. RGC somas (arrowheads) and axons (arrows) that lack CTB are found in both groups. Scale: 30 μm. (**d**) Coronal sections (top rows) of the SC following intravitreal injection of CTB (green) into eyes of vehicle- and BIRB 796-treated mice. Transport deficits are indicated by dotted lines. OD: optic disc representation (arrows). Retinotopic maps (bottom rows) reconstructed from serial sections of SC with optic disc gap indicated (circles). Density of the transported CTB signal ranges from 0% (blue) to 50% (green) to 100% (red). Medial (M) and rostral (R) orientations are indicated. Scale bars: 500 μm. (**e**) Scatter plot showing level of intact transport to the SC from vehicle- and BIRB 796-treated DBA/2 J mice. Thin black lines indicate mean ± SEM. Data expressed as mean ± SEM. Statistical comparisons made using two-sided t-tests. n = 20 mice per group (**a** and **b**); n = 28 SCs for vehicle-treated group and 30 SCs for BIRB 796-treated group (**d** and **e**).

DBA/2 J mice have mutations in two genes, *Gpnmb* and *Tyrp1*, which cause pigment dispersion and iris atrophy within the anterior chamber, resulting in increasing IOP with age^37,48–51^. Baseline IOP was similar for vehicle-treated mice and BIRB 796-treated mice (15.7 ± 0.1 and 15.6 ± 0.1 mm Hg, respectively; p = 0.8). IOP was significantly elevated (20.2%) compared to baseline IOP (red dotted line, Fig. 4a) in vehicle- and BIRB 796-treated mice by 5 months of age and remained elevated for the next 16 weeks (p < 0.001). Mean treatment IOP (Fig. 1a) in vehicle-treated mice (19.5 ± 1.0 mm Hg) and BIRB 796-treated mice (19.5 ± 0.9 mm Hg) increased 42.4% compared to respective baseline IOPs (Fig. 4b, p < 0.001). Treatment with BIRB 796 had no effect on mean treatment IOP compared to vehicle treatment (p = 0.9).

Colocalization of fluorescently labeled CTB and phosphorylated neurofilament-heavy (pNF-H) in RGC somas and axons in vehicle-treated and BIRB 796-treated DBA/2 J mice varied within groups from robust uptake and localization in axons to no uptake by RGCs and little to no localization in axons (Fig. 4c). Similarly, anterograde transport of CTB to the SC of vehicle-treated and BIRB 796-treated DBA/2 J mice varied from nearly complete to entirely absent (Fig. 4d, top panels). Quantification of CTB transport in the SC showed intact transport in vehicle-treated mice averaged 32.0 ± 6.8%, with a range of 89.8 to 0.8% (Fig. 4e). BIRB 796 treatment did not protect axonal transport to the SC in BIRB 796-treated mice (28.8 ± 6.0%, range of 95.9 to 0.3%) when compared to vehicle-treated mice (p = 0.7).

### BIRB 796 did not protect ganglion cell axons in DBA/2 J mice

**Figure 5:**
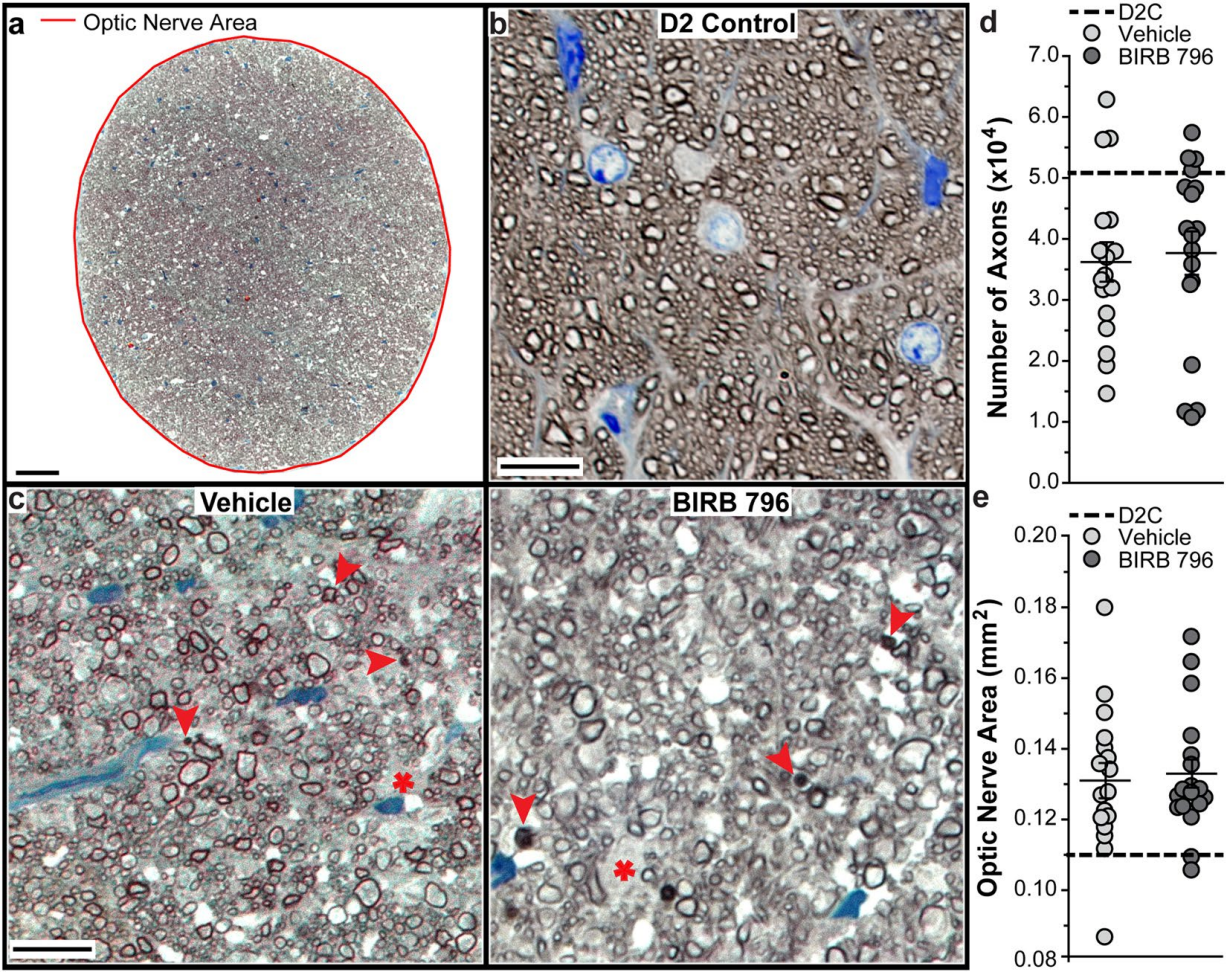
BIRB 796 and RGC axons in DBA/2 J optic nerve. (**a**) Representative image of DBA/2 J mouse optic nerve montage used to count RGC axons using AxonJ^46^. Red line indicates optic nerve area as measured using Fiji^45^. Scale: 50 μm. (**b**) Representative image from a DBA/2J-Gpnmb+/SjJ (D2 Control) mouse optic nerve montage. Scale: 10 μm. (**c**) Representative images from vehicle- and BIRB 796-treated DBA/2 J mouse optic nerve montages. Degenerating axon profiles (arrowheads) and glial hypertrophy (asterisk) are indicated. Scale: 10 μm. (**d**) Scatter plot showing RGC axon number from vehicle- and BIRB 796-treated DBA/2 J mouse optic nerve. Thin black lines indicate mean ± SEM. Thick black dotted line indicates D2 Control RGC axon number for non-statistical comparison. (**e**) Scatter plot showing optic nerve area from vehicle- and BIRB 796-treated DBA mouse optic nerve. Thin black lines indicate mean ± SEM. Thick black dotted line indicates D2 Control RGC axon number for non-statistical comparison. Data expressed as mean ± SEM. Statistical comparisons made using two-sided t-tests. n = 8 optic nerve montages for D2 Control, 17 optic nerve montages for vehicle-treated and 18 optic nerve montages for BIRB 796-treated groups.

A representative DBA/2 J optic nerve montage used in counting RGC axons within the entire montage is shown in Fig. 5a. We measured optic nerve cross-sectional area (indicated by red line) in whole nerve montages using Fiji. Representative images of optic nerves from vehicle-treated and BIRB 796-treated DBA/2 J mice (Fig. 5c) showed disorganized axon bundles, degenerating axon profiles (arrowheads) and glial hypertrophy (asterisk) when compared to a 10-month-old DBA/2J-Gpnmb+/SjJ (D2 Control) optic nerve (Fig. 5b). Axon number ranged from 14,756 to 62,961 axons in vehicle-treated nerves and from 10,846 to 57,545 axons in BIRB 796-treated nerves. These counts are similar to previously published axons counts for DBA/2 J mice indicative of a range of degenerative states (~10,000 to 65,000 axons)^46,52^. BIRB 796 did not protect mean axon number in BIRB 796-treated nerves (37,696 ± 3,559 axons) when compared to vehicle-treated nerves (36,231 ± 3,235 axons; p = 0.4, Fig. 5d), nor did it effect cross-sectional nerve area (0.131 ± 0.005 mm^2^ versus 0.133 ± 0.004 mm^2^; p = 0.7, Fig. 5e).

### BIRB 796 effectively reduces levels of p38 MAPK-related targets in DBA/2 J mice

**Figure 6:**
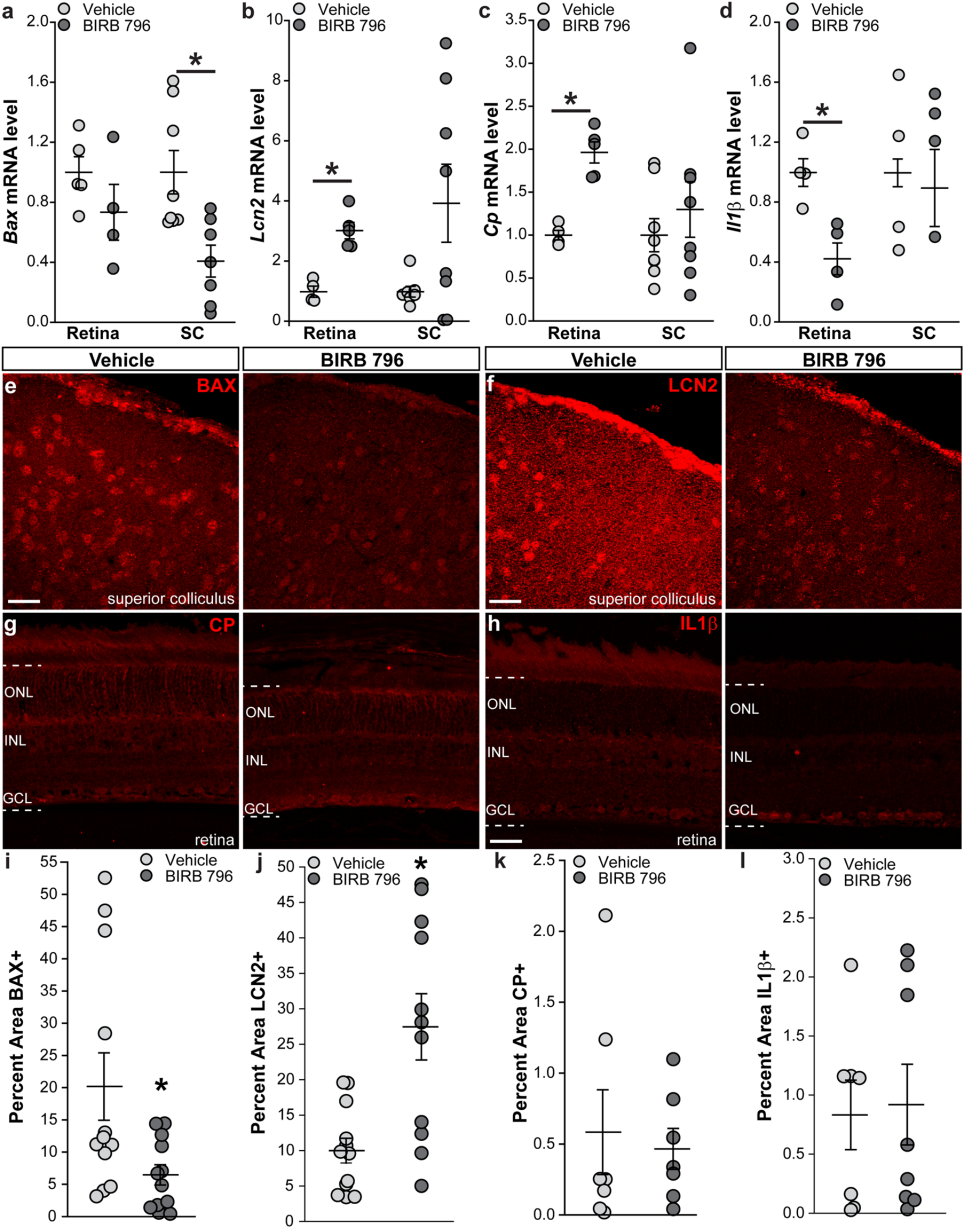
BIRB 796 modulates expression of p38-related injury targets in DBA/2 J mice. (**a**) Quantitative RT-PCR measurements of mRNA encoding Bcl2-associated X protein (*Bax*). *p = 0.01. (**b**) Quantitative RT-PCR measurements of mRNA encoding lipocalin-2 (*Lcn2*). *p = 0.0006. (**c**) Quantitative RT-PCR measurements of mRNA encoding ceruloplasmin (*Cp*). *p = 0.0003. (**d**) Quantitative RT-PCR measurements of mRNA encoding interleukin-1β (*Il1*β). *p = 0.007. (**e**) Confocal images from vehicle- and BIRB 796-treated DBA/2 J superior colliculus showing immunolabeling for BAX (red). Scale bars: 30 μm. (**f**) Confocal images from vehicle- and BIRB 796-treated DBA/2 J superior colliculus showing immunolabeling for lipocalin-2 (LCN2, red). (**g**) Confocal images from vehicle- and BIRB 796-treated DBA/2 J retina showing immunolabeling for ceruloplasmin (CP, red). Dotted lines indicate retinal region where label was quantified. NFL: nerve fiber layer; GCL: ganglion cell layer; IPL: inner plexiform layer; INL: inner nuclear layer; OPL: outer plexiform layer; ONL: outer nuclear layer. (**h**) Confocal images from vehicle- and BIRB 796-treated DBA/2 J retina showing immunolabeling for interleukin-1β (IL1β, red). Dotted lines indicate retinal region where label was quantified. NFL: nerve fiber layer; GCL: ganglion cell layer; IPL: inner plexiform layer; INL: inner nuclear layer; OPL: outer plexiform layer; ONL: outer nuclear layer. (**i**) Scatter plot showing percent area of vehicle- and BIRB 796-treated DBA/2 J superior colliculus immunolabeled for BAX. Thin black lines indicate mean ± SEM. *p = 0.02. (**j**) Scatter plot showing percent area of vehicle- and BIRB 796-treated DBA/2 J superior colliculus immunolabeled for lipocalin-2. Thin black lines indicate mean ± SEM. *p = 0.001. (**k**) Scatter plot showing percent area of vehicle- and BIRB 796-treated DBA/2 J retina immunolabeled for ceruloplasmin. Thin black lines indicate mean ± SEM. (**l**) Scatter plot showing percent area of vehicle- and BIRB 796-treated DBA/2 J retina immunolabeled for interleukin-1β. Thin black lines indicate mean ± SEM. Data expressed as mean ± SEM. Statistical comparisons made using two-sided t-tests. n = 5 vehicle-treated and 4 BIRB 796-treated retinas, 8 vehicle-treated and 7 BIRB 796-treated SCs (**a**); n = 4 vehicle-treated and 5 BIRB 796-treated retinas, 7 vehicle-treated and 8 BIRB 796-treated SCs (**b**)*;* n = 4 vehicle-treated and 5 BIRB 796-treated retinas, 8 vehicle-treated and 8 BIRB 796-treated SCs (**c**); n = 4 vehicle-treated retinas, BIRB 796-treated retinas, vehicle-treated SCs and BIRB 796-treated SCs (**d**); n = 12 vehicle-treated and 12 BIRB 796-treated SCs (**e,i**); n = 12 vehicle-treated and 11 BIRB 796-treated SCs (**f,j**); n = 7 vehicle-treated and 7 BIRB 796-treated retinas (**g,k**); n = 7 vehicle-treated and 8 BIRB 796-treated retinas (**h,l**).

We examined the levels of p38-related injury targets in the retina and SC of vehicle- and BIRB 796-treated DBA/2 J mice to determine if BIRB 796 reached our target tissue. Quantitative RT-PCR measurements showed a 59% reduction in mRNA encoding Bcl2-associated X protein (*Bax*) within the SC of BIRB 796-treated mice compared to vehicle-treated mice (p = 0.01, Fig. 6a). A similar reduction was seen in the retina however it was not significant (p = 0.22). *Lipocalin-2* mRNA levels increased 203% (p = 0.0006, Fig. 6b) and *ceruloplasmin* increased 96% (p = 0.0003, Fig. 6c) in BIRB-treated retinas compared to vehicle-treated retinas while *interleukin 1β (IL1β)* decreased 57% in BIRB-treated retinas compared to vehicle-treated retinas (p = 0.007, Fig. 6d). Treatment with BIRB 796 had no effect on mRNA levels of these genes within the SC (p ≥ 0.08). Examining protein expression in SCs and retinas from DBA/2 J mice showed reduced immunolabeling for BAX (Fig. 6e) and lipocalin-2 (Fig. 6f) within BIRB-treated SCs compared to vehicle-treated SCs. In contrast, immunolabeling for ceruloplasmin (Fig. 6g) and interleukin 1β (Fig. 6h) in BIRB 796-treated retinas appeared similar to vehicle-treated retinas. Quantification of BAX levels in vehicle- and BIRB 796-treated SCs showed a 68% reduction in BAX levels in BIRB 796-treated SCs (p = 0.02, Fig. 6i). We observed a decrease of 64% in lipocalin-2 levels in BIRB 796-treated SCs when compared to vehicle-treated SCs (p = 0.001, Fig. 6j). Quantification of ceruloplasmin (Fig. 6k) and interleukin 1β (Fig. 6l) revealed BIRB treatment had no effect on protein levels compared to vehicle (p ≥ 0.7).

### BIRB 796 did not protect anterograde transport following microbead injection in squirrel monkeys

**Figure 7:**
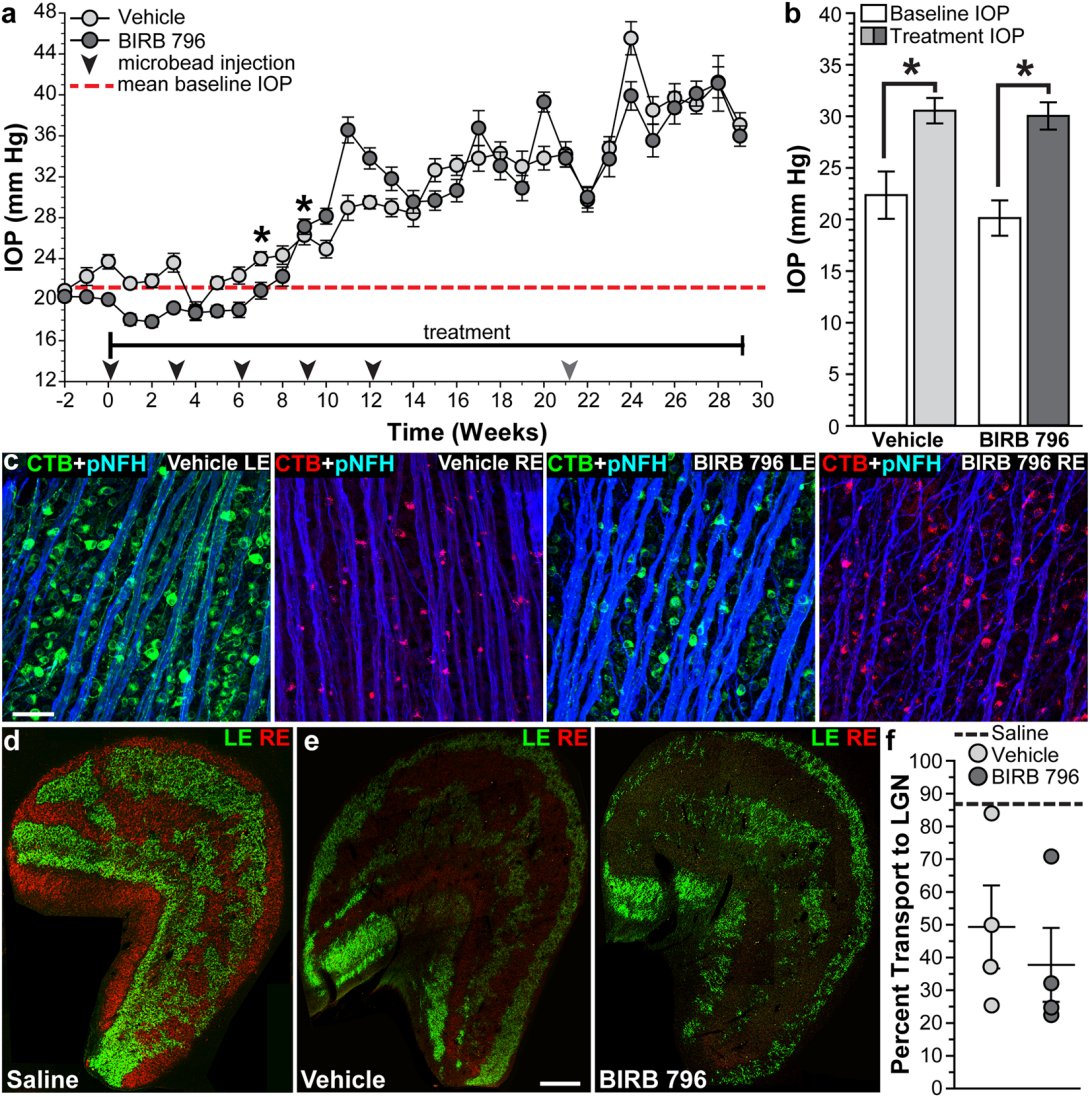
BIRB 796 and anterograde transport in squirrel monkeys following IOP elevation. (**a**) Mean intraocular pressure (IOP) in squirrel monkeys (SMs) following bilateral injection of microbeads into the anterior chamber of vehicle- and BIRB 796-treated SMs. Arrowheads indicate microbead injections. Red dashed line indicates mean baseline IOP from all SMs. *p ≤ 0.016 versus baseline IOP. (**b**) Bar graph showing mean baseline IOP and treatment IOP for vehicle- and BIRB 796-treated SMs. *p ≤ 0.02. (**c**) Confocal images of whole-mounted retinas showing RGC uptake and transport of CTB (green in left eyes, red in right eyes) in vehicle- and BIRB 796-treated SMs. Phosphorylated neurofilament-heavy (pNF-H; blue) expression in RGC somas and axons is shown. Scale: 50 μm. (**d**) Coronal section of a lateral geniculate nucleus (LGN) from a saline-injected SM from a previous study^39^. CTB transported from the left eye (LE, green) and the right eye (RE, red) are shown. Scale: 500 μm. (**e**) Coronal sections of LGN from a vehicle-treated and a BIRB 796-treated SM. CTB transported from the left eye (LE, green) and the right eye (RE, red) are shown. Scale: 500 μm. (**f**) Scatter plot showing percent intact transport to the LGN from microbead-injected eyes from vehicle- and BIRB 796-treated SMs. Thin black lines indicate mean ± SEM. Thick black dotted line indicates transport to the LGN from saline-injected eyes (Saline) from a previous study for non-statistical comparison^39^. Data expressed as mean ± SEM. Statistical comparisons made using two-sided t-tests. n = 4 eyes per group (**a**,**b**); n = 4 LGNs for vehicle-treated group and 4 LGNs for BIRB 796-treated group (**e**,**f**).

Baseline IOP measurements in SMs ranged from 20.0 ± 0.4 to 23.7 ± 0.7 mm Hg with a mean of 21.3 ± 0.5 mm Hg and were similar for vehicle-treated and BIRB 796-treated SMs (p = 0.4). IOP was significantly elevated compared to baseline IOP (red dotted line, Fig. 7a) in vehicle-treated SMs after three microbead injections and in BIRB 796-treated SMs after four microbead injections; IOP remained elevated for 20–22 weeks (p ≤ 0.016). Mean treatment IOP (Fig. 1a) increased 36.6% compared to baseline IOPs (22.4 ± 2.3 mm Hg; p = 0.02, Fig. 7b) in vehicle-treated SMs (30.5 ± 1.2 mm Hg) and 49.1% to 30.0 ± 1.3 mm Hg in BIRB 796-treated SMs compared to baseline IOP (20.1 ± 1.7 mm Hg; p = 0.003, Fig. 7b). Treatment with BIRB 796 had no effect on mean treatment IOP compared to vehicle treatment (p = 0.8).

Unlike rodents, where nearly all RGC axons terminate in contralateral targets, RGC axons in SMs terminate to ipsilateral and contralateral targets in equal (50:50) proportion^53^. To examine RGC anterograde transport in SMs, we injected CTB-488 into left vitreous chambers and CTB-594 into right vitreous chambers (Fig. 1b) and quantified CTB transport to the lateral geniculate nucleus (LGN), the primary ganglion cell subcortical target in primates as previously described^39^. We verified RGC uptake and initial transport of CTB in whole-mounted retinas from vehicle-treated and BIRB 796-treated SMs (Fig. 7c). Colocalization of CTB and phosphorylated neurofilament-heavy (pNF-H) was similar in vehicle-treated and BIRB 796-treated SM retinas. Representative images of LGNs from vehicle-treated and BIRB 796-treated SMs (Fig. 7e) showed reduced anterograde transport due to elevated IOP when compared to the LGN of a SM that received saline injection instead of microbeads (Fig. 7d). Quantification of anterograde transport to the LGN (Fig. 7f) showed elevated IOP due to microbead injection reduced transport 43% to 49.3 ± 12.7% intact transport in vehicle-treated SMs and 57% to 37.7 ± 11.3% intact transport in BIRB 796-treated SMs. Treatment with BIRB 796 did not protect transport to the LGN when compared to vehicle treatment (p = 0.5). We observed similar transport deficits to the SC of vehicle-treated and BIRB 796-treated SMs; BIRB treatment did not protect transport to the SC (p = 0.3).

### BIRB 796 did not protect ganglion cell axons following microbead injection in squirrel monkeys

**Figure 8:**
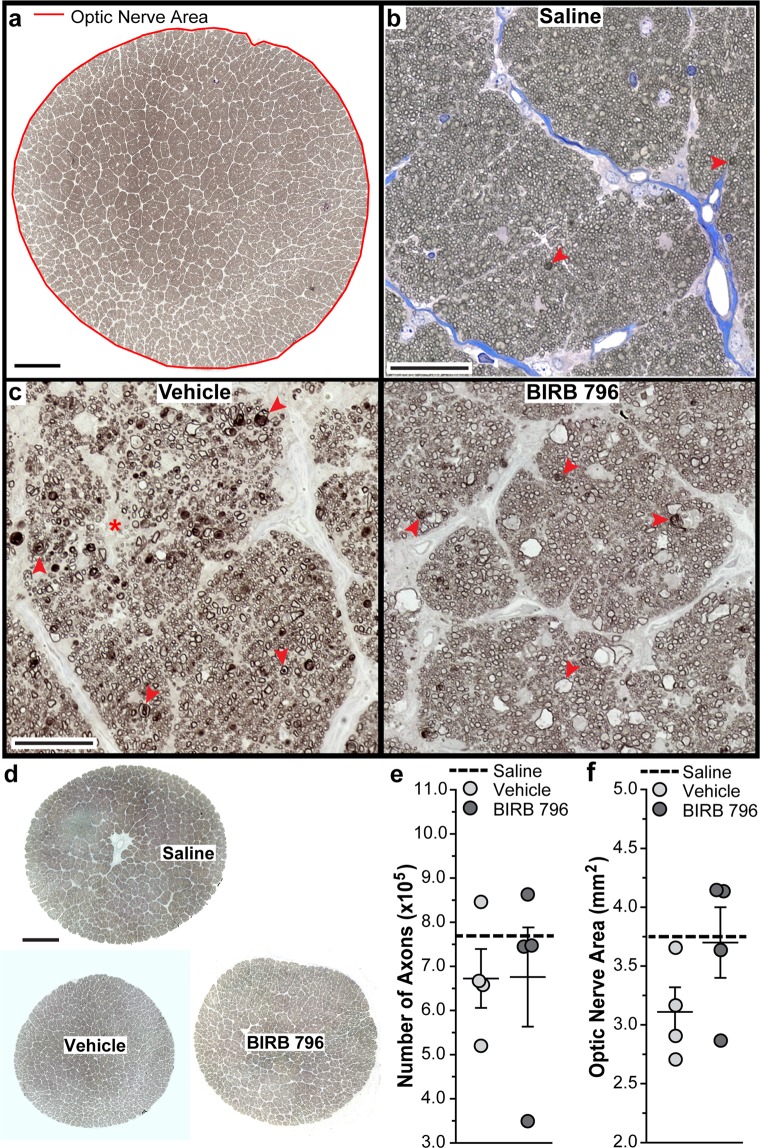
BIRB 796 and RGC axon survival in squirrel monkey optic nerves following IOP elevation. (**a**) Representative image of SM optic nerve montage used to count RGC axons using AxonJ^46^. Red line indicates optic nerve area as measured using Fiji^45^. Scale: 250 μm. (**b**) Representative image from saline-injected SM optic nerve montage from a previous study^39^. Scale: 100 μm. (**c**) Representative images from vehicle- and BIRB 796-treated SM optic nerve montages. Degenerating axon profiles (arrowheads) and disorganized axon fascicle (asterisk) are shown. Scale: 100 μm. (**d**) Low magnification images of saline-injected SM optic nerve, vehicle-treated SM optic nerve and BIRB 796-treated SM optic nerve. Scale: 500 μm. (**e**) Scatter plot showing RGC axon number from vehicle- and BIRB 796-treated SM optic nerves. Thin black lines indicate mean ± SEM. Thick black dotted line indicates mean RGC axon number from saline-injected eyes (Saline) from a previous study for non-statistical comparison^39^. (**f**) Scatter plot showing area from vehicle- and BIRB 796-treated SM optic nerves. Thin black lines indicate mean ± SEM. Thick black dotted line indicates mean optic nerve axon area from saline-injected eyes (Saline) from a previous study for non-statistical comparison^39^. Data expressed as mean ± SEM. Statistical comparisons made using two-sided t-tests. n = 4 optic nerve montages for vehicle-treated and 4 optic nerve montages BIRB 796-treated groups.

A representative SM optic nerve montage used in counting RGC axons within the entire montage is shown in Fig. 8a. We measured optic nerve cross-sectional area (indicated by red line) in whole nerve montages using Fiji. Representative images of optic nerves from vehicle-treated and BIRB 796-treated SMs (Fig. 8c) showed numerous degenerating axon profiles, disorganized fascicles, and glial scarring, indicating axon degeneration and astrocyte hypertrophy when compared to when compared to saline eyes from a previous study (Fig. 8b)^54,55^. Comparing low magnification images of optic nerves from vehicle-treated and BIRB 796-treated SMs (Fig. 8d) to a nerve from a saline-injected eye showed vehicle-treated nerves appeared smaller than BIRB-796 treated nerves and saline nerves. Axon number ranged from 519,823 to 845,851 axons in vehicle-treated SM nerves and from 348,917 to 862,845 axons in BIRB 796-treated nerves. BIRB 796 did not protect mean axon number in BIRB 796-treated nerves (675,905 ± 112,427 axons) when compared to vehicle-treated nerves (672,596 ± 66,836 axons; p = 0.4, Fig. 8e). Optic nerve area decreased ~17% in vehicle-treated nerves (3.11 ± 0.21 mm^2^) when compared to BIRB 796-treated nerves (3.70 ± 0.3 mm^2^) but was not significant (p = 0.2, Fig. 8f).

### BIRB 796 detected in vitreous and reduced activation of p38 MAPK downstream targets in squirrel monkey retina

**Figure 9:**
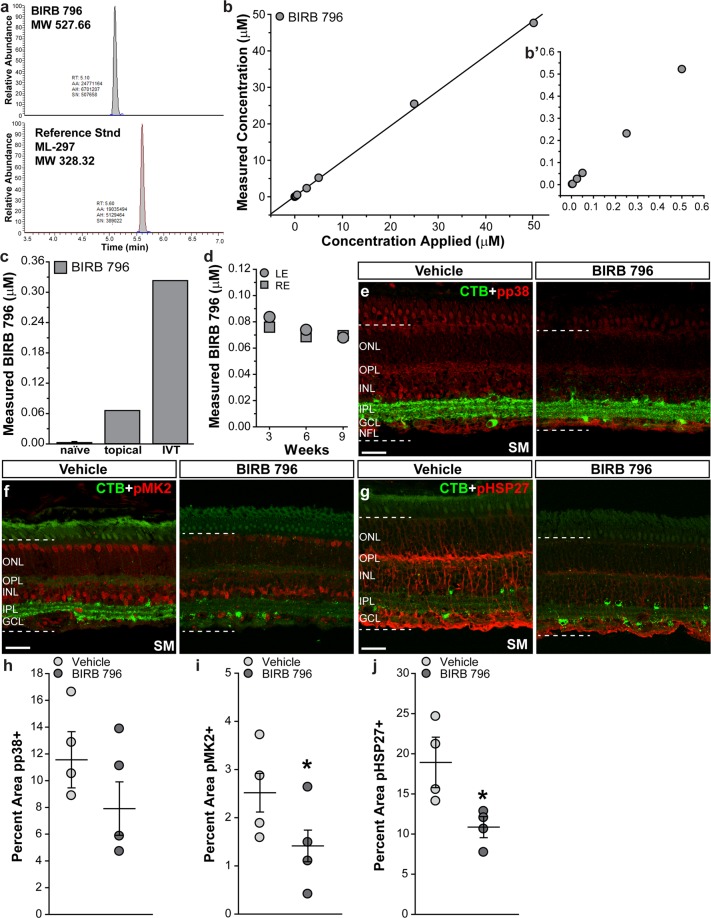
BIRB 796 detected in SM vitreous and attenuated activation of p38 MAPK pathway members in SM retinas. (**a**) Relative abundance of BIRB 796 and reference standard ML-297 in vitreous samples measured by liquid chromatography–mass spectrometry (LC-MS). (**b**) Scatter plot showing LC-MS measurements of BIRB 796 concentration (μM) in SM vitreous samples with known concentrations of BIRB 796 added. (**b’**) shows nM range of BIRB 796 concentrations applied. (**c**) Bar graph showing LC-MS measurement of BIRB 796 in SM vitreous following topical delivery of 3% BIRB 796 (40 μl/day) to cornea for one week, or two intravitreal injections of 0.75% BIRB 796 (40 μl) one week apart. Last topical dose and last intraviteal injection given just prior to vitreous sampling. (**d**) Scatter plot showing LC-MS measurements of BIRB 796 concentration (μM) in SM vitreous samples following topical delivery of 3% BIRB 796 (40 μl/day) to cornea for 9 weeks. Vitreous sampled every 3 weeks. (**e**) Confocal images from vehicle- and BIRB 796-treated SM retina showing immunolabeling for phosphorylated p38 MAPK (pp38; red). CTB uptake (green) by RGCs is shown. Dotted lines indicate region where label was quantified. GCL: ganglion cell layer; IPL: inner plexiform layer; INL: inner nuclear layer; OPL: outer plexiform layer; ONL: outer nuclear layer. Scale bars: 30 μm. (**f**) Confocal images from vehicle- and BIRB 796-treated SM retina showing immunolabeling for phosphorylated MK2 (pMK2; red). CTB uptake (green) by RGCs is shown. Dotted lines indicate region where label was quantified. GCL: ganglion cell layer; IPL: inner plexiform layer; INL: inner nuclear layer; OPL: outer plexiform layer; ONL: outer nuclear layer. (**g**) Confocal images from vehicle- and BIRB 796-treated SM retina showing immunolabeling for phosphorylated heat shock protein 27 (pHSP27; red). CTB uptake (green) by RGCs is shown. Dotted lines indicate region where label was quantified. GCL: ganglion cell layer; IPL: inner plexiform layer; INL: inner nuclear layer; OPL: outer plexiform layer; ONL: outer nuclear layer. (**h**) Scatter plot showing percent area of vehicle- and BIRB 796-treated SM retinas immunolabeled for phosphorylated p38 MAPK (pp38). Thin black lines indicate mean ± SEM. (**i**) Scatter plot showing percent area of vehicle- and BIRB 796-treated SM retinas immunolabeled for phosphorylated MK2 (pMK2). Thin black lines indicate mean ± SEM. *p = 0.04. (**j**) Scatter plot showing percent area of vehicle- and BIRB 796-treated SM retinas immunolabeled for phosphorylated heat shock protein 27 (pHSP27). Thin black lines indicate mean ± SEM. *p = 0.03. Data expressed as mean ± SEM. Statistical comparisons made using two-sided t-tests. n = 6 SM eyes for naïve, 1 SM eye for topical and 1 SM eye for intravitreal (**c**); n = 2 SM eyes (**d**); n = 4 eyes for vehicle-treated and 4 eyes for BIRB 796-treated groups (**e–j**).

We collaborated with the Vanderbilt Mass Spectrometry Research Center to measure drug content in SM vitreous samples using liquid chromatography–mass spectrometry (LC-MS). To determine the LC-MS signature for BIRB 796 and construct a calibration curve, we added known concentrations of BIRB 796 and ML-297, a reference standard, to SM vitreous humor samples and used LC-MS measure the ratio of peaks. The LC-MS signatures of BIRB 796 and ML-297 are shown in Fig. 9a. We found that detection of BIRB 796 in SM vitreous is remarkably linear and is predicted well by a broad range of applied concentrations from 2 nM up to 50 μM (Fig. 9b). Using this methodology, we detected measurable quantities of BIRB 796 within the linear range in vitreous humor samples from one SM that received two intravitreal injections of BIRB 796 (0.75%) in one eye and from one SM that received daily topical application of BIRB 796 (3%) for one week (Fig. 9c). As expected, the intravitreal injection produced a 6-fold higher concentration than topical dosing. We also detected stable levels of BIRB 796 in the vitreous of one SM after daily topical application of BIRB 796 (3%) for 9 weeks, with vitreous samples collected every 3 weeks (Fig. 9d). These data suggest BIRB 796 was able to penetrate into the eye when applied topically to the SM cornea. Next, we examined levels of phosphorylated p38 MAPK, phosphorylated MK2 and phosphorylated Hsp27 in retinas from vehicle- and BIRB 796-treated SMs to determine if BIRB 796 acts within our target tissue^25,27^. Phosphorylated p38 MAPK (pp38, Fig. 9e) appeared to localize to astrocytes and Műller glia end-feet in the NFL and to CTB + RGC somas in the GCL in vehicle- and BIRB-treated SM retinas. In vehicle-treated retinas, phosphorylated p38 also localized to the INL and to processes in the outer plexiform layer (OPL); this labeling appeared reduced in BIRB 796-treated retinas. Immunolabeling for phosphorylated MK2 (Fig. 9f) localized to CTB + RGCs and other nuclei in the GCL and to nuclei in the INL in vehicle- and BIRB-treated SM retinas. Treatment with BIRB 796 appeared to reduce pMK2 levels. Phosphorylated HSP27 (Fig. 9g) localized to Műller cell bodies and end-feet in vehicle- and BIRB 796-treated retinas. Again, treatment with BIRB 796 appeared to reduce pHSP27. Quantification showed BIRB 796 lowered phosphorylated p38 levels 29.8% in BIRB 796-treated retinas compared to vehicle-treated retinas (Fig. 9h); however, this was not significant (p = 0.2). Phosphorylated MK2 levels were reduced 43.8% in BIRB 796-treated retinas compared to vehicle-treated retinas (p = 0.04, Fig. 9i) and phosphorylated HSP27 levels decreased by 42.7% in BIRB 796-treated retinas compared to vehicle-treated retinas (p = 0.03, Fig. 9j).

## Discussion

We examined the neuroprotective effects of BIRB 796 in the DBA/2 J mouse and in rats and squirrel monkeys following microbead occlusion (summarized in Table 1). Daily topical treatment with BIRB 796 had no effect on IOP in any model but did protect anterograde transport to the rat SC and RGC axon degeneration in the rat optic nerve after 4 weeks of IOP elevation (Figs. 2 and 3). Unfortunately, treatment with BIRB 796 for 24 weeks in DBA/2 J mice or 29 weeks in SMs had no effect on anterograde transport to RGC targets or RGC axon survival in the optic nerve even though it decreased levels of p38 downstream targets in both models and was detected in SM vitreous after treatment. While data presented here implicate p38 MAPK in glaucomatous progression, BIRB 796 treatment for extended experimental periods did not protect RGC structure or function in the genetic and inducible glaucoma models used.

**Table 1:** Study Summary.

Feature	Rat	DBA/2 J Mice	Squirrel Monkey
Age of animals	7–9 months	10 weeks	2–3 years
Method IOP elevation	microbead	genetic mutations	microbead
Size/vol. microbeads/injx.	15 μm/5 μl	n/a	35 μm/40 μl
Duration of IOP elevation	4 weeks	16 weeks	20–22 weeks
Mean IOP elevation	34% (vehicle)	42% both	37% (vehicle)
	36% (BIRB 796)		49% (BIRB 796)
Duration of treatment	4 weeks	24 weeks	29 weeks
BIRB 796 Dosage	2–2.5 mg/kg	5.0 mg/kg	1.5 mg/kg
Adverse Effects	none noted	none noted	none noted
Volume CTB injected	2 μl/eye	1 μl/eye	40 μl/eye
Anterograde Transport	↑ 80% vs. vehicle*	N.C. vs. vehicle	N.C. vs. vehicle
Axon Degeneration	8% ↓ vs. vehicle**	N.C. vs. vehicle	N.C. vs. vehicle

injx: injection. N.C.: no change. *p = 0.00007 and **p = 0.007 compared to vehicle microbead.

One potential reason for the lack of efficacy in the SM and DBA/2 J models presented here is drug availability in the ocular compartment following topical dosage. We did not perform pharmacokinetics or measure the levels of BIRB 796 in ocular tissues in rats or DBA/2 J mice, but we did detect BIRB 796 in SM vitreous samples after topical dosing for 9 weeks. Twice daily topical dosing in rats protected anterograde transport and RGC axons after 4 weeks of elevated IOP suggesting BIRB 796 was able to penetrate the ocular compartment and exert effects in rat retinas and/or optic nerves. Daily topical dosing over 24 plus weeks of elevated IOP in DBA/2 J mice and SMs did not protect RGC structure and function, but we did observe reduced expression of p38-related targets in treated DBA/2 J retinas and superior colliculi, and decreased activation of MK2 and Hsp27 in treated SM retinas. This would suggest BIRB 796 did reach the retina in DBA/2 J mice and SMs but failed to protect RGC axonal structure and function over longer treatment intervals.

Although BIRB 796 availability within the eye could account for the decreased efficacy observed in this study, a transient effect of p38 inhibitors has been observed previously. Due to the role of p38 MAPK in chronic inflammation, many p38 inhibitors have been developed and have advanced to clinical trials^32^. However, no p38 inhibitor has progressed past this phase and into market due to poor clinical efficacy, lack of sustained therapeutic effects, or adverse or off-target effects^26,27,32,56,57^. For example, Weisman *et al*. assessed VX-745 treatment in patients with rheumatoid arthritis (RA)^58^. Although the drug showed clinical efficacy and appeared well tolerated by patients over the 12 week treatment, VX-745 was withdrawn from clinical trials due to neurological adverse effects observed in a long-term animal trial^27,57^. A similar study using VX-702 treatment for RA showed no statistically significant difference between VX-702 and placebo treatment after 12 weeks, and reductions in inflammatory markers that were observed after one week of treatment had returned to baseline values by week 4^59^. Cohen *et al*. examined the efficacy of the p38 inhibitor pamapimod in patients with RA^60^. Following 12 weeks of treatment, fewer patients had a clinical response to pamapimod when compared to the standard treatment for RA, higher doses of pamapimod showed twice the incidence of adverse effects, and reductions in inflammatory markers were observed early in the trial but were not sustained. Over the course of the trial, Cohen *et al*. found no decrease in plasma levels of pamapimod suggesting lack of efficacy was not due to metabolism of the drug^60^. In a 24-week trial of SCIO-469 in patients with RA, Genovese *et al*. found no significant differences in clinical efficacy when compared to placebo, and elevated liver enzymes were detected in patients treated at the higher dose of SCIO-469^61^. Much like previous trials, initial decreases in inflammatory markers returned to baseline levels prior to study end^61^. Similar results have been observed in clinical trials of p38 inhibitors in Crohn’s disease, cardiovascular disease, chronic obstructive pulmonary disease, cancer, and neurotic pain, where clinical efficacy was not achieved or sustained, and/or the study terminated due to adverse events^62–68^. Our results mirror those from clinical trials in that positive outcomes were not sustained over long treatment intervals. The most likely explanation for this may be the target itself.

The p38 MAPK pathway is activated by cellular stress and injury, making it an attractive target for therapeutic intervention in many diseases^26^. However, p38 activation can stimulate anti-inflammatory cytokines (e.g. IL-10) in addition to pro-inflammatory cytokines like TNFα and IL-1; hence inhibiting p38 MAPK in hopes of suppressing inflammation may actually activate inflammatory responses^56,69^. Further complicating the use of p38 inhibitors is evidence suggesting p38 MAPK can regulate cellular differentiation, migration, tissue homeostasis, and the cytoskeleton^70,71^. Inhibiting p38 from activating downstream targets that are anti-inflammatory and/or contribute to homeostasis could explain the poor efficacy observed in clinical trials. Another issue that could contribute to the lack of efficacy and/or a sustained effect of p38 inhibitors may be pathway regulation. Activation of p38 MAPK can occur via p38-specific signals, more general MAPK signals, or by MAPK kinase-independent signals where p38 autophosphorylates after binding accessory proteins or is phosphorylated at non-canonical sites^25,70,72^. These MAPK kinase-independent signals could result in inhibitors not binding all p38 MAPK present in the target tissue, leaving the pathway active. The MAPK pathway relies on feed-back loops for fine tuning, and inhibiting p38 could result in hyperactivation of upstream activating MAPK kinases, resulting in activation of other MAPK pathways^56,73,74^. For example, Jones *et al*. recently showed that p38 inhibition in synovial fibroblast cultures resulted in activation of NFκB, JNK, and MEK signaling in the presence of inflammatory cytokines^75^. This crosstalk suggests these signaling pathways are interconnected and may share common signals, such as CREB or phosphatases. Inhibition of JNK or MEK also caused activation of p38 signaling, but to a lesser degree than p38 inhibition had on those pathways^75^. Pathway crosstalk could be decoupled in the assay used by Jones *et al*., either by using multiple MAPK inhibitors, or by targeting kinases upstream of p38 or downstream substrates^75^. Much is known about MAPKs, yet cell- and tissue-specific responses to and the regulation of p38 MAPK in various contexts complicate its utility as a therapeutic target. Further work is needed to better understand how to modulate this pathway for effective clinical outcomes that can be sustained over long treatment periods without adverse effects.

## Methods

### Animals

The Vanderbilt University Institutional Animal Care and Use Committee approved all experimental procedures, which meet the standards and guidelines set forth in the Animal Welfare Act and the Guide for the Care and Use of Laboratory Animals, Eighth Edition. We obtained male Brown Norway rats (7–9 months old) from Charles River Laboratories (Wilmington, MA) and randomly placed them into naïve (n = 4), vehicle (n = 9) or BIRB 796 (n = 9) groups. We obtained male DBA2/J mice (10 weeks of age) and DBA/2J-Gpnmb+/SjJ (D2 Control) from The Jackson Laboratory (Bar Harbor, ME). DBA2/J mice were randomly placed into vehicle (n = 20) or BIRB 796 (n = 20) groups. D2 Control mice (n = 4) were aged to 10 months. Rodents were maintained in a 12-hour light-dark cycle with standard rodent chow available *ad libitum*. We obtained male Bolivian squirrel monkeys (*Saimiri boliviensis;* 2–3 years of age) with no history of diabetes or head/eye trauma from the University of Texas MD Anderson Cancer Center (Bishop, TX). Squirrel monkeys (SMs) were socially housed, received environmental enrichment daily, and were maintained in a 12-hour light-dark cycle with standard primate biscuits and water available *ad libitum*. SMs were randomly placed into a vehicle (n = 2 SMs, 4 eyes) or BIRB 796 (n = 2 SMs, 4 eyes) group. No SM in either group showed signs of impairment or distress (itching or rubbing of the eye, eye closure, dehydration, Anorexia, lacerations, bites, or scratch wounds, abnormal gait or posture, head tilt, lethargy, swellings or growths, vomiting or diarrhea, or difficulty breathing) during the study. SMs from a previous cohort (n = 4) were used for BIRB 796 bioavailability studies (see below).

### Intraocular pressure measurements

We measured intraocular pressure (IOP) bilaterally in awake rats (n = 9 per group) and in anesthetized (2.5% isoflurane) DBA/2 J mice (n = 20 per group) using a Tono-Pen XL rebound tonometer (Medtronic Solan, Jacksonville, FL) as previously described^17,24^. We measured IOP bilaterally in awake SMs (n = 4 eyes per group) using a Tono-Pen XL, a restraint tube, and positive-reinforcement training techniques as previously described^39^. We performed IOP measurements 2–3 times weekly in rodents and weekly in SMs following administration of 0.5% proparacaine hydrochloride ophthalmic solution (Patterson Veterinary Supply, Inc.) as a local anesthetic. IOP measurements began at least one week prior to treatment and concluded at least 48 hours prior to sacrifice. Naïve rats (n = 4) had one IOP measurement prior to CTB injection.

### Microbead intracameral injection

In rats, we elevated IOP unilaterally by injecting 5.0 μl of 15 μm polystyrene microbeads (Molecular Probes, Eugene, OR) into the anterior chamber^38^. The fellow eye received an equivalent volume of saline to serve as an internal control. Rats (n = 9 per group) received one microbead or saline injection. Naïve rats (n = 4) did not receive a saline or microbead injection in either eye. We elevated IOP bilaterally in SMs by injecting 40 μl of 25–35 μm polystyrene microbeads (FP-30052-5; Spherotech, Inc., Lake Forest, IL) plus 1% Hydroxypropyl Methyl Cellulose (HPMC) in sterile phosphate buffered saline (PBS) into the anterior chamber using a 31-gauge needle attached to a syringe (Fisher Scientific) as described previously^39^. SMs (n = 2 per group, 4 eyes per group) received six microbead injections/eye over the 29-week treatment period.

### BIRB 796 Preparation, treatment, and intraocular bioavailability

A 3% drug suspension of BIRB 796 (LC Laboratories, Woburn, MA) was prepared by adding 30 mg of BIRB 796 to 1 ml buffer [0.75% NaCl, 0.5% Hydroxypropyl methylcellulose (HMPC) viscosity 2600–5600 cP (Sigma Aldrich, St. Louis, MO), 0.5% sodium phosphate dibasic decahydrate (Fisher Scientific, Waltham, MA), 0.01% benzalkonium chloride (Sigma) in sterile water, pH 7.2, sterile filtered]. The suspension was vortexed for 30 seconds, placed in a water bath sonicator (Elma S30H, Elma Schmidbauer GmbH) and sonicated for 1 minute, and then vortexed again for 30 seconds. We repeated this process until no large pieces of drug were observed upon visual inspection of suspension. We prepared fresh vehicle and BIRB 796 suspensions twice a week (every 2 to 3 days). Vehicle (buffer with no BIRB 796 added) and BIRB 796 suspension were stored at 4 °C and kept on ice during dosing. Rats (n = 9 per group) received vehicle or BIRB 796 (10 μl per eye) applied topically to the cornea with a pipette twice daily, Monday through Friday, beginning at time of microbead injection and continuing for 4 weeks. Naïve rats (n = 4) did not receive a vehicle or BIRB 796 treatment. Mice (n = 20 per group) received BIRB 796 or vehicle (5 μl per eye) applied topically to the cornea with a pipette once daily, Monday through Friday, beginning at 12 weeks of age and continuing for 24 weeks. SMs (n = 2 per group, 4 eyes per group) received BIRB 796 or vehicle (40 μl per eye) applied topically to the cornea with a pipette once daily, Monday through Friday, beginning at time of microbead injection and continuing for 29 weeks. The dosing strategy used here resulted in compliance rates of 75% (21 doses of a possible 28) in rats, 71.4% (120 doses out of a possible 168) in mice, and 70.4% (143 doses out of a possible 203) in SMs. This level of compliance is similar to that of glaucoma patients using pressure-lowering medications (~70%)^76–80^. Animals were observed daily by authors or by Vanderbilt Division of Animal Care technicians for signs of distress and/or physical impairment. We also monitored changes in ocular tissues (e.g. opacity of the cornea, irritation of the conjunctiva).

For BIRB 796 bioavailability studies, we collected vitreous humor (50 μl/eye) from untreated SMs (n = 3 SMs, 6 eyes) by preparing, anesthetizing and monitoring SMs as described for Microbead Intracameral Injection previously^39^. We washed the eyes in sterile saline solution, and provided pre-emptive anesthesia using 0.5% proparacaine drops. We withdrew vitreous humor from the intravitreal cavity using a 31-gauge needle attached to a syringe and stored samples in sterile Fisherbrand 2.0 ml Microcentrifuge Tubes with Locking Snap Cap (Fisher Scientific) at −20 °C. Reversal of anesthesia and post-procedure monitoring were described previously^39^. While under anesthesia, one SM received 0.75% BIRB 796 (40 μl) via intravitreal injection into one eye using a 31-gauge needle attached to a syringe. A second intravitreal injection of BIRB 796 was performed into the same eye one week later, immediately before a second vitreous collection. Another SM from this group received 3% BIRB 796 (40 μl) applied topically to the cornea of one eye daily for one week. The third SM received no BIRB 796. One week after initial vitreous collection, we collected vitreous humor (50 μl/eye) from all SM eyes (n = 6) and stored samples at −20 °C. One SM (n = 1, 2 eyes) was treated bilaterally with 3% BIRB 796 (40 μl/eye) daily for 9 weeks; vitreous humor (50 μl/eye) was collected every 3 weeks. No SM in this cohort (n = 4) had more than three anesthetic events to collect vitreous humor or inject BIRB 796.

We collaborated with the Vanderbilt Mass Spectrometry Research Center with the goal of using liquid chromatography–mass spectrometry (LC-MS) to measure drug content in SM vitreous samples. ML297 (≥98%; Cayman Chemical, Ann Arbor, MI) was used as a reference standard. HPLC-grade acetonitrile (300 μl; JT Baker; VWR Scientific) was added to vitreous samples (100 μl), vortexed and centrifuged to remove any precipitated proteins or other particulates. Supernatant (300 μl) was removed, dried and reconstituted in 100 μl of the mobile phases of the LC (see below). We used a Surveyor HPLC system (Thermo-Fisher, Waltham, MA) consisting of a quaternary pump/degasser, refrigerated autosampler, and column heater to perform sample analyses. For chromatographic separations, we used a Poroshell 120 HPLC column (3.0 mm × 50 mm, 2.7 μm) (Agilent Technologies, Santa Clara, CA) equipped with an Acquity UPLC in-line stainless steel filter unit (0.2 μm, Waters Corp, Milford, MA). We set the column compartment temperature to 40 °C, and the autosampler tray temperature to 4 °C. Mobile phases were made up of 0.1% (v/v) formic acid (Fluka, 98%; Sigma-Aldrich. St. Louis, MO) in (A) HPLC-grade H2O (JT Baker; VWR Scientific) and in (B) HPLC-grade acetonitrile (JT Baker; VWR Scientific). We used the following gradient conditions: 0–1 min, B = 5%; 1–7 min, B = 5–95%; 7–9 min, B = 95%; 9.01 min, B = 5%; 9.01–11 min, B = 5%. The flow rate was maintained at 300 μL/min. The total chromatographic run time was 15 min. The sample injection volume was 10 μL. The autosampler injection valve and the sample injection needle were flushed with 400 μL methanol/water (2:1) containing 1% acetic acid between each injection. We performed tandem mass spectrometric detection using a TSQ Quantum triple-stage quadrupole mass spectrometer (Thermo-Fisher, Waltham, MA) equipped with a standard API-1 electrospray source and a 100 μm ID stainless steel capillary^81^. The instrument was tuned and calibrated every four to six weeks over a mass range of m/z 182 to m/z 997 with a mixture of tyrosine peptides using the manufacturer’s autotune procedure. The mass spectrometer was operated in positive ion mode. Quantitation was based on multiple reaction monitoring (MRM) detection of BIRB 796: m/z 528 → 256, CE 31 V and ML-297: m/z 329 → 200, CE 18 V. The following optimized parameters were used for the detection of analyte and internal standard: N2 sheath gas 40 psi; N2 auxiliary gas 5 psi; spray voltage 4.0 kV; capillary temperature 300 °C; capillary offset 35 V, tube lens offset 105 V, Ar collision gas 1.5 mtorr; scan time 100 ms; Q1/Q3 peak width at half-maximum 0.7 m/z. We performed data acquisition and quantitative spectral analysis using Thermo-Finnigan Xcalibur version 1.3 and Thermo-Finnigan LCQuan version 2.7, respectively. We constructed calibration curves by plotting peak area ratios (BIRB 796/ML-297) against BIRB 796 concentrations for a series of eight vitreal standards (3 nM – 50 μM). Each calibration level was processed in duplicate in order to determine the precision of the assay. A weighting factor of 1/C was applied in the linear least-squares regression analysis to maintain homogeneity of variance across the concentration range.

### RNA extraction and quantitative RT-PCR

Mice (n = 5 for vehicle, n = 5 for BIRB 796) were sacrificed by cervical dislocation and retinas quickly extracted from the eye cups. We extracted total RNA from each retina (n = 10 per group) or SC (n = 10 per group) according to the SV Total RNA Isolation System kit protocol (Promega). RNA concentration and purity were determined using a NanoDrop 8000 (Thermo Scientific, Wilmington, DE). mRNA was then reverse-transcribed into cDNA using the 1st Strand cDNA Synthesis System for quantitative RT-PCR (Origene) following the manufacturer’s instructions. Reaction mixtures were diluted 3-fold and subjected to qRT-PCR amplification using AB 7300 Real Time PCR System (Applied Biosystem) and FastStart SYBR Green Master mix (Roche). Primers are listed in Table 2. Cycling conditions were the same for all primer sets: 94 °C for 2 minutes and 40 cycles of 94 °C for 30 seconds, 60 °C for 30 seconds, and 72 °C for 30 seconds. For each primer set, a dissociation curve was performed to confirm a single peak corresponding to a single product and no primer dimer. Relative product quantities for each transcript were performed in triplicate, normalized to GAPDH mRNA as an endogenous control, and determined using the comparative ΔCT method^82^.

**Table 2:** Primers for Quantitative RT-PCR.

Name	Forward (5′-3′)/Reverse (5′-3′)	Reference
Bax	AAGCTGAGCGAGTGTCTCCGGCG/GCCACAAAGATGGTCACTGTCTGCC	^84^
Ceruloplasmin	CTGATGTCTTTGACCTTTTCCCTG/TTCTCGTTTTCCACTTATCGCC	^85^
Interleukin-1β	ATGCCTTCCCCAGGGCATGT/GCCCATCAGAGGCAAGGAGGA	^86^
Lipocalin-2	ACTGAATGGGTGGTGAGTGTGG/TCTGGCAACAGGAAAGATGGAG	^85^
GAPDH	AGGTCGGTGTGAACGGATTTG/GGGGTCGTTGATGGCAACA	^87^

### CTB injection and anterograde axonal transport analysis

We anesthetized rats (n = 8 for vehicle, 9 for BIRB 796) and mice (n = 14 for vehicle, 15 for BIRB 796) with 2.5% isoflurane and intravitreally injected 2 µl (rat) or 1 µl (mice) of a 1% solution of cholera toxin subunit B (CTB) conjugated to Alexa Fluor-488 (Molecular Probes, CA) per eye as previously described^18^. Forty-eight hours later we perfused rodents transcardially with phosphate buffered saline (PBS) followed with 4% paraformaldehyde in PBS. We anesthetized SMs (n = 2 per group) using ketamine (Patterson Veterinary) plus dexmedetomidine (Patterson Veterinary) and maintained anesthesia with 2.5% isoflurane before intravitreally injecting 40 μl of a 1% solution of CTB-488 (left eyes) or CTB-594 (right eyes) using 31-gauge needle attached to a syringe^39^. We euthanized SMs using sodium pentabarbitol (Euthasol; Patterson Veterinary) via intravenous injection and transcardially perfused with PBS followed by 4% paraformaldehyde in PBS. Brains were cryoprotected in 20% sucrose/PBS (rodents) and 30% sucrose/PBS (SMs) and coronal midbrain sections (50–52 µm) cut on a Leica SM 2000R freezing sliding microtome (Leica Microsystems, Vienna, Austria) with a freezing stage (Brain Research Laboratories, Newton MA) and 250 mm flat back permanent microtome knife (C.L. Sturkey, Inc., Lebanon PA). We placed alternating sections of SC (rodents and SMs) and LGN (SMs) on Fisherbrand Superfrost Plus Microscope Slides (Fisher Scientific) and coverslipped with Fluoromount G (Southern Biotech, Birmingham, AL). We imaged rodent SC sections using a Nikon Ti Eclipse microscope at 4×(Nikon Instruments Inc., Melville, NY) and the intensity of CTB signal was quantified using a custom ImagePro macro (Media Cybernetics, Bethesda, MD) as previously described^18^. For SMs, we imaged SC and LGN sections using an Olympus FV-1000 inverted confocal microscope at 10x through the Vanderbilt University Medical Center Cell Imaging Shared Resource. We quantified the intensity of CTB-488 or CTB-594 signal in the SC and LGN using a custom ImagePro macro (Media Cybernetics, Bethesda, MD) after normalizing to background^18^. We calculated the percent of intact transport for each section as previously described^39^. We verified CTB uptake by RGCs in retinas from all groups using an Olympus FV-1000 inverted confocal microscope.

### Optic nerve analysis

A 3 mm section of optic nerve (n = 8 nerves for naïve rat, vehicle-saline and vehicle-microbead rats, 9 nerves for BIRB 796-saline and BIRB 796-microbead rats; n = 8 nerves for D2 control, 17 nerves for vehicle-treated and 18 nerves for BIRB 796-treated DBA/2 J mice; n = 4 nerves vehicle-treated and 4 nerves BIRB 796-treated SMs) proximal to the globe was isolated, post-fixed for 1 hour in 4% paraformaldehyde (rodents) or 2 hours in 2% glutaraldehyde (SMs), and prepared for embedding and semi-thin cross-sectioning as described previously^83^. We incubated optic nerve segments in 2% osmium tetroxide (Electron Microscopy Sciences, Hatfield, PA) in 0.1 M sodium cacodylate buffer (Electron Microscopy Sciences) for 1 hour then dehydrated samples in a graded ethanol series. We embedded optic nerve segments in Araldite 502 and Embed Resin 812 (Electron Microscopy Sciences). We collected optic nerve cross sections (0.7 μm) using a Leica Ultra Microtome EM UC7 (Leica Microsystems) and stained with 1% paraphenylenediamine (PPD; in a 1:1 mixture of methanol and 2-propanol) and 1% toluidine blue to identify myelin sheaths and glia, respectively. We imaged sections using 10x and 40×(SMs) or 100×(rodents) oil-immersion and differential interference contrast optics as a whole optic nerve montage with a microscope equipped with a motorized X-Y-Z stage and a digital SLR camera (Nikon H600L and DS-Ri2). We counted RGC axons within the entire optic nerve montage using the AxonJ macro in Fiji^45,46^ and measured ON cross-sectional area using Fiji.

### Immunohistochemistry and image quantification

Whole eyes dissected from perfused rodents were prepared as whole-mount retinas (n = 4 retinas for naïve rats, n = 5 retinas for vehicle-saline and vehicle-microbead rats, 5 retinas for BIRB 796-saline and BIRB 796-microbead rats; n = 10 retinas for vehicle-treated and 10 retinas for BIRB 796-treated DBA/2 J mice), paraffin-processed (n = 8 eyes for vehicle-treated and 8 eyes for BIRB 796-treated DBA/2 J mice), or stored for later use (n = 10 eyes for vehicle-treated and 12 eyes for BIRB 796-treated DBA/2 J mice). For paraffin processing, we post-fixed eyes in 4% paraformaldehyde for 2 hours to overnight, then transferred to cassettes. We dehydrated eyes in a graded ethanol series followed by xylene clarification and three changes of paraffin (StatLab, McKinney TX) infusion before the final paraffin embedding. We collected sections (6 μm) using a Leica Ultra Microtome RM2255 (Leica Microsystems, Vienna, Austria). We dissected SM whole eyes (n = 4 eyes for vehicle-treated and 4 eyes for BIRB 796-treated) and bisected the retina, preparing one-half for whole-mount immunohistochemistry (n = 4 half-retinas for vehicle-treated and 4 half-retinas for BIRB 796-treated) and the other half for cryoembedding (n = 4 half-retinas for vehicle-treated and 4 half-retinas for BIRB 796-treated). We cryoprotected retinas in 10–30% sucrose/PBS followed by a 1:1 solution of 30% sucrose/PBS + Fisher Healthcare Tissue Plus OCT Compound (Fisher Scientific). We cryoembedded eyes in Fisher Healthcare Tissue Plus OCT Compound and collected sections (10 μm) using a CryoStar NX50 cryostat (ThermoFisher) and MX35 Premiere+ Microtome blades (ThermoFisher). We performed immunohistochemistry on paraffin sections, cryosections, and whole-mount retinas as previously described^17,24^. We blocked non-specific antibody binding with 5% normal donkey serum (Jackson ImmunoResearch Laboratories, Inc., West Grove, PA), 0.1% Triton-X 100 (Sigma-Aldrich) and PBS + 0.02% sodium azide (Fisher Scientific). We diluted primary antibodies (Table 3) in 3% normal donkey serum, 0.1% Triton-X 100 and PBS + 0.02% sodium azide and incubated with sections or whole-mount retinas overnight at room temperature or 72 hours at 4 °C. Following three washes (15 minutes each) with PBS + 0.02% sodium azide, we incubated sections or whole-mount retinas with appropriate secondary antibodies (Table 3) 2 hours at room temperature or overnight at 4 °C. Following three washes (15 minutes each) with PBS + 0.02% sodium azide, we placed whole-mounted retinas on Fisherbrand Superfrost Plus Microscope Slides (Fisher Scientific) and coverslipped whole-mounted retinas, paraffin sections or cryosections with DAPI Fluoromount G (paraffin and cryosections) or Fluoromount G (whole-mounted retinas) (Southern Biotech, Birmingham, AL).

**Table 3:** Antibodies Used for Immunohistochemistry.

Antigen	Dilution	Catalog #	Vendor
Bax	1:200	bs-3010R	1
Ceruloplasmin (CP)	1:500	#611488	2
Interleukin-1β (IL1β)	1:50	AF-501-NA	3
Lipocalin-2 (LCN2)	1:200	bs-1373R	1
Phosphorylated Hsp27	1:100	#2406	4
Phosphorylated MAPKAPK-2 (pMK2)	1:50	#3007	4
Phosphorylated neurofilament-H (pNF-H)	1:1000	801601	5
Phosphorylated p38 MAPK (pp38)	1:1000	#9216 S	4
Cy3 Donkey Anti-Mouse IgG (H + L)	1:200	715–166–150	6
Alexa Fluor 647 Donkey Anti-Mouse IgG (H + L)	1:200	715–606–150	6
Alexa Fluor 488 Donkey Anti-Rabbit IgG (H + L)	1:200	711-546-152	6
Cy3 Donkey Anti-Rabbit IgG (H + L)	1:200	711-166-152	6

1: Bioss Antibodies, Inc., Woburn, Massachusetts; 2: BD Transductions Labs, San Jose, CA; 3: R&D Systems, Minneapolis, MN; 4: Cell Signaling Technology, Danvers, MA; 5: BioLegend, San Diego, CA; 6: Jackson ImmunoResearch Laboratories, Inc., West Grove, PA.

We imaged whole-mounted retinas using an Olympus FV-1000 inverted confocal microscope at 60 × (rodents) and 40×(SMs). For quantification, we imaged SC and retinal sections using an Olympus FV-1000 inverted confocal microscope at 60× resulting in images that were 512 × 512 pixels, 211.968 × 211.968 μm. Identical microscope settings were used to acquire images for quantification for a given protein of interest. We captured images in the mid-peripheral region of the retina for all vertical sections. One image per SC section or retinal vertical section was captured per DBA/2 J mouse (n = 12 for SC, n = 8 for retina). Two images were captured for each SM retinal vertical section, one on either side of the optic disc in the mid-peripheral region. Label was quantified in both images and the mean was used for quantification (n = 4 for retina for each group). A naïve observer quantified immunolabel in images using a custom macro in ImagePro (Media Cybernetics) that determines the percent area of the positive label^18^. Areas quantified for a protein of interest in SC or retinal sections did not differ between groups (p ≥ 0.2 for DBA/2 J, p ≥ 0.17 for SMs).

### Statistical analysis

All data are expressed as mean ± standard error of the mean (SEM) unless indicated otherwise. The number of samples used in each experiment is provided in the appropriate methods section and figure legend. Statistical comparisons between two independent measurements were made using two-sided t-tests, following confirmation of normality for each using the Shapiro-Wilk normality test; samples for which normality failed were compared using the Mann-Whitney Rank Sum Test (SigmaPlot 14.0, Systat Software, Inc., Chicago, IL). Comparisons between multiple groups were made using one-way analysis of variance (ANOVA) followed by the Holm-Sidak Pairwise Multiple Comparison test (SigmaPlot 14.0) as described previously^39^.

## Data Availability

All data generated or analyzed during this study are included in this published article; certain data have been reproduced for comparison where indicated from Lambert, W.S., Carlson, B.J., Ghose, P. *et al*. Towards A Microbead Occlusion Model of Glaucoma for a Non-Human Primate. Sci Rep 9, 11572 (2019) 10.1038/s41598-019-48054-y.
